# Identifying potential barriers and solutions to patient partner compensation (payment) in research

**DOI:** 10.1186/s40900-022-00341-1

**Published:** 2022-02-23

**Authors:** Dawn P. Richards, Kelly D. Cobey, Laurie Proulx, Shoba Dawson, Maarten de Wit, Karine Toupin-April

**Affiliations:** 1 Five02 Labs Inc, Toronto, ON Canada; 2 Canadian Institutes of Health Research Institute of Musculoskeletal Health and Arthritis, University of British Columbia, Vancouver, BC Canada; 3 Canadian Arthritis Patient Alliance, Ottawa, ON Canada; 4 Patient Research Partner, Toronto, ON Canada; 5 Meta-Research and Open Science Program, University of Ottawa Heart Institute, Ottawa, ON Canada; 6 School of Epidemiology and Public Health, University of Ottawa, Ottawa, ON Canada; 7 Patient Research Partner, Ottawa, ON Canada; 8 Bristol Medical School, University of Bristol, Bristol, UK; 9 Patient Research Partner, Zaltbommel, Netherlands; 10 School of Rehabilitation Sciences, University of Ottawa, Ottawa, ON Canada; 11 Department of Pediatrics, University of Ottawa, Ottawa, ON Canada; 12 Children’s Hospital of Eastern Ontario Research Institute, Ottawa, ON Canada; 13 Institut du Savoir Montfort, Ottawa, ON Canada

**Keywords:** Patient engagement, Patient and public involvement, Patient partner, Patient research partner, Payment, Compensation, Institutional barriers, Barriers

## Abstract

Research that engages patients on the research team is often supported by grant funding from different organizations and, in some cases, principal investigators (who control the grant funding) provide patient partners with compensation (or payment) for their contributions. However, we have noted a gap in resources that identify and address barriers to compensating patient partners (no matter the size, degree or length of their engagement). In this paper, we present thoughts and experiences related to barriers to compensating patient partners with the goal of helping individuals identify and find solutions to these obstacles. Based on our experiences as individuals who live with chronic conditions and are patient partners, and those who are researchers who engage patient partners, we have identified eight barriers to compensating patient partners. We discuss each of these barriers: lack of awareness about patient partnership, institutional inflexibility, policy guidance from funders, compensation not prioritized in research budgets, leadership hesitancy to create a new system, culture of research teams, preconceived beliefs about the skills and abilities of patient partners, and expectations placed on patient partners. We demonstrate these barriers with real life examples and we offer some solutions. To further demonstrate these barriers, we ask readers to reflect on some scenarios that present realistic parallel situations to those that patient partners face. The intention is to illustrate, through empathy or putting yourself in someone else’s shoes, how we might all do better with respect to institutional barriers related to patient partner compensation. Last, we issue a call to action to share resources and identify actions to overcome these barriers from which we will create an online resource repository.

## Plain English summary

Research teams that engage patients as partners sometimes provide compensation or payment to these patient partners for their contributions. There does not seem to be many resources that identify and tackle barriers to compensating patient partners. Based on our experiences as people who live with chronic conditions and who are patient partners, and as researchers who engage patient partners, we have identified eight barriers to compensating patient partners. We discuss each of these barriers: lack of awareness about patient partnership, institutional inflexibility, policy guidance from funders, compensation not prioritized in research budgets, leadership hesitancy to create a new system, culture on research teams, preconceived beliefs about patient partners’ skills and abilities, and expectations placed on patient partners. We illustrate these with real-life scenarios and we offer some solutions. Last, we issue a call to action to the global community that engages patient partners in their research teams to share resources and identify actions to overcome these barriers. By doing so, we can begin to create an online resource repository and help patient partners and researchers focus on working together rather than wasting energy navigating a system that is not well adapted to engaging patient partners.

## Background

For some health researchers, research teams and organizations, patient and public involvement in research is becoming the de facto way they carry out their projects [1–6]. Patient and public involvement in research, sometimes also called patient engagement or patient-oriented research, is “research being carried out ‘with’ or ‘by’ members of the public rather than ‘to’, ‘about’ or ‘for’ them," [7] or simply put, when patients are partners on the research team [8]. Patient engagement in research has been encouraged, and in many cases, supported through funding by research organizations such as the National Institute for Health Research [9], the Patient-Centered Outcomes Research Institute [10] and the Canadian Institutes of Health Research [11] (organizations that each use slightly different terms related to patient engagement). As this approach becomes normalized by research teams, early adopters face a number of challenges regarding its implementation. One important consideration relates to patient partners’ compensation.

In this paper, we use the term “patient partner” to denote “individuals with personal experience of a health issue [or condition] and informal caregivers, including family and friends” [12] and who are members of the research team (no matter the size or duration of a research project or initiative), not to denote participants in research [13]. The topic of this manuscript may also apply to public or citizen contributors in research, however as our experiences are predominantly with patient partners, we prefer and will use this latter term. We use the term ‘compensation’ to refer to payment of salary, wages, honorarium, or resources to build capacity/skill with respect to this engagement; it can be used interchangeably with payment [14]. Compensation or payment of patient partners is separate from the process of reimbursing their expenses to be part of the research team (e.g., paying parking or travel expenses for a patient to attend a meeting) [13, 15].

While some resources exist (e.g., journal articles, online guidance from organizations) about compensating patient partners, many of them relate to building a case for, explaining the concept and principles of payment of patient partners, how to have a conversation about this topic, concrete suggested amounts for compensation, and highlighting general implications (often tax- or social benefits-related) of receiving compensation [13, 15–19]. Compensation to patient partners is crucial to ensure equity in the team, helps recognize different motivations for patients being on the team, respects vulnerability that patient partners may bring to their work, can facilitate commitment and ability to contribute to the team, and removes barriers to participation on a team [15]. An in-depth exploration of whether or not all patient partners wish to accept compensation as well as specific forms or methods of compensation are beyond the scope of this publication.

Despite the rationale for compensating patient partners, barriers often exist in practical implementation that may significantly impact the relationship between the research team, institution or organization, and patient partners. For example, barriers to enable compensation can lead to patient partners feeling unvalued and unappreciated, and thinking that the energy related to compensation may not be worth the efforts they are investing in the research project. This may lead to them abandoning their wish to be compensated and/or them leaving the research project altogether. Researchers may feel like they are fighting an uphill battle when they face these barriers, and may therefore not feel inclined to include patient partners on their team for future projects. Researchers and administrators may both feel that unnecessary time and budget is being spent on simply processing or ‘papering’ patient partner compensation instead of focused on “doing” the research. Overall, these barriers further contribute to unconsciously reinforcing the power imbalance that already exists when patient partners are part of a research team [15, 18]. For these reasons, among others, we feel it is important to have a larger conversation about barriers to patient partner compensation.

We have identified a gap in resources that specifically identifies and addresses barriers to compensating patient partners. We provide personal perspectives as people who live with chronic, lifelong disease who work actively on numerous research projects and researchers who have incorporated patient involvement in their projects. We present our collective thoughts and experiences related to barriers to compensating patient partners. This paper explores and discusses: (1) institutional and research-related cultural barriers to compensating patient partners, and potential solutions and resources to overcome these barriers; (2) scenarios and reflective points to help illustrate institutional barriers to compensation for those who are not patient partners; and (3) a call to action for the international community to share resources and identify actions to overcome these barriers, leading to creating an online resource repository. These barriers are presented in a manner that is relatable for patient partners, other members of the research team, and those who enable patient partner compensation at research and other institutions. While we primarily share our experiences related to academic, hospital, or other not for profit organizations, some of these challenges may also apply to for-profit organizations (e.g., pharmaceutical companies) that engage patient partners. Our overall goal is to help people who are part of the ‘enterprise’ of engaging patient partners in research to identify and seek solutions to these barriers, in the short and long term.

## Main text

### Barriers to patient partner compensation

**Fig. 1:**
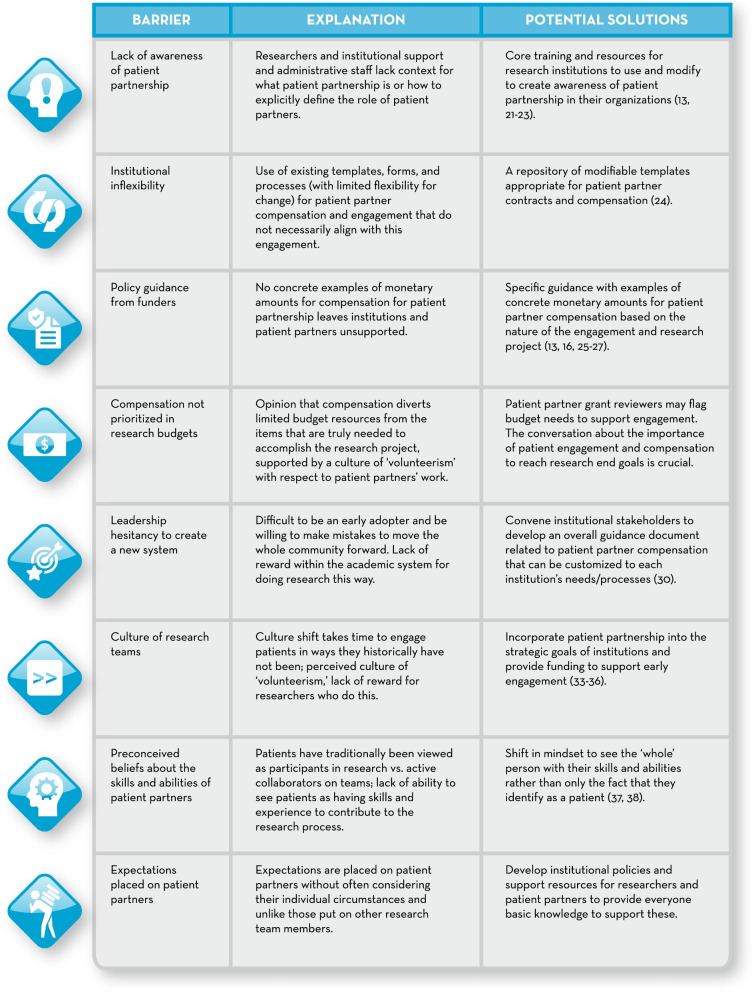
Barriers to patient partner compensation and potential solutions. These are some frequently experienced barriers to providing patient partners with compensation, and explanations of and some potential solutions and resources to address these barriers

In this section, we present what we have frequently experienced as institutional barriers to providing patient partners with compensation and include illustrative examples and potential solutions and resources. Examples are presented anonymously so no individual or organization is identified. However, it is important to support the barriers we identify with real and concrete examples. Further, these are not presented in any order of priority or importance (see Fig. 1 for a summary of this section).

#### Lack of awareness about patient partnership

This barrier relates to staff who manage, facilitate, and/or process compensation for patient partners. These staff are often not familiar with the concept of or lack the context for patient partnership or its possibility when they are called upon. These staff may include the research support and administrative staff (for example, finance, human resources, contracts, etc.) who are involved in processing and ‘papering’ the arrangement related to patient partner compensation. Researchers also often have limited context on how to explicitly define the role of patient partners in their research project that could help others understand why compensation is appropriate and/or necessary.

Some examples that illustrate this barrier:

- A request from the finance department for the researcher to send the Research Ethics Board (REB) approval letter to compensate a patient partner. The finance department assumed that the patient partner was a research participant and wanted to ensure the REB had approved payment for participation.
- A lack of clarity on how researchers should handle the process of compensation. Many researchers feel they have to help create new processes when they make this type of request since there is no clear guidance put in place by institutions. Often researchers are creating these forms and processes on their own due to lack of standards and institutional support.
- In some jurisdictions, a lack of clear and consistent guidance from institutions about when there are tax implications or tax paperwork is required in relation to patient partner payment. For example, in Canada there is a $500 payment amount, after which tax paperwork is required (though all payment should be claimed) [20]. Some institutions issue tax paperwork before this limit and others do not and policies may be incorrectly designed around these tax provisions. Patient partners may also not be informed about these procedures.
- Patient partners being set up in finance systems with external collaborator or university supplier agreements which do not properly recognize their contributions and require a tremendous amount of paperwork and a laborious process.

A potential solution to address this barrier is to create awareness of patient partnership in organizations and to develop and recommend mandatory core training and resources (including templates) for research institution personnel (e.g., finance, contracts, and human resources staff). For example, the Canadian Institutes of Health Research describes the differences between patients as research partners and as research participants [13, 21]; this material could be re-purposed to address institutional training needs. It is also possible that some institutions have developed processes and templates that are applicable to patient partner compensation. Sharing these between institutions will help decrease barriers. The Dutch Federation of Patient organizations (PF) has developed a guide regarding the compensation of patient representatives. It contains information about the maximum amount that patients may receive without paying taxes (180 € per month to a maximum of 1800 € per year) and provides recommended amounts per hour (100 €) for patients attending research committee meetings [22, 23]. Many institutions and individuals know these rules, and policies are adjusted around these amounts. For example, some patient partners receive benefits from the government and some institutions are flexible about compensating (e.g., backdating payments) to meet the rules of these state benefit payments.

#### Institutional inflexibility

This barrier relates to institutions’ lack of ability to change or be flexible to change and may stem from the expectation that patient partner needs and circumstances will simply fit within the existing processes and template forms traditionally used by institutions. However, since these processes and forms/templates are usually used to pay employees or to pay for other “services” from institutions, for-profit vendors or companies, they do not necessarily fit patient partners’ needs. Thus, institutional finance and human resource policies often do not align with engaging individual patient partners or individuals from community groups. It appears to be difficult for organizations to adapt processes and forms/templates to the needs of this specific community. Institutional financial and human resources policies have been built for collective purposes related to agreements, policies, and processes. There appears to be a limited amount of discretion with which staff are empowered in this area. Further, many patient partners are unaware or reluctant to push back on standard, boiler plate processes and templates that are not appropriate for their engagement. These processes can be intimidating and not conducive to creating trusting relationships from the start of an engagement.

Examples that illustrate this barrier:

- Intimidating and lengthy legal contracts that include legal language, jargon and clauses on indemnification and insurance which far outweigh compensation amount, and which are most likely not reflective of the potential ‘risk’ of having a patient partner involved on the research team.
- The request for patient partners to have their own liability insurance for contract purposes. This is reflective of requirements for a vendor or contractor and is not likely reflective of risk involved with having a patient partner on the team, nor is it even easy or affordable for patient partners to obtain liability insurance.
- Requesting a procurement process that requires competitive bids from patient partners to be submitted with respect to applying to patient partner work. The notion of pitting potential patient partners against one another in a competitive bidding process does not foster inclusion or help meet the goals of patient involvement in research.

Potential solutions to help address this barrier include co-creating with patient partners or having a repository of modifiable templates appropriate for patient partner contracts, human resources and finance policy templates, and billing and resources that support institutional administration in identifying what resources may need to be modified to support patient partnership. There are few existing resources in this space, though some developed for patients and patient organizations involved in working relationships with the pharmaceutical industry may be helpful as a start [24]. For some patient partners, compensation may not be monetary and may instead relate to helping them build their own capacity to engage. In the UK, honorary contracts offer a way for patient partners to have access to an institutional email address, library, short courses, and other resources that might not otherwise be possible. These identified resources and potential approaches should not replace the possibility of monetary compensation entirely, but can be considered as part of improving the capacity of patient partners to contribute to research, and should be shared with patient partners as an option.

#### Policy guidance from funders

This barrier relates to research funders using ‘soft’ language or language that is open to interpretation by institutions and individuals rather than providing concrete policy guidance about patient partner compensation. The ability for interpretation of language around compensation is likely due to a desire to provide flexibility to different institutions based on their own processes, however it also unintentionally leaves institutions unsupported in facilitating patient partnership and leaves patient partners unsupported in advocating for fair compensation.

Some examples that illustrate this barrier include advocacy for patient partner compensation by various organizations but without clear policy guidance:

- The Canadian Institutes of Health Research indicates that patient partners may be provided with compensation for their roles on research teams in their principles document [13].
- The Patient-Centered Outcomes Research Institute has a principles based document [19].
- The European Alliance of Associations for Rheumatology (formerly the European League Against Rheumatism) has recommendations for the inclusion of patient representatives in scientific projects that include language about payment being an important aspect of patient partner contribution (recommendation 8), but at the time of publication in 2011, indicated that “No consensus could be reached in our task force meetings and supportive literature is weak. Further research is necessary before an appropriate recommendation on payment can be made.”[1].

Potential resources to help address this barrier are documents from Canada, the US and the UK that include specific potential monetary amounts to consider for compensation based on project requirements and potential involvement [16, 25–27]. Resources that specify when compensation is not appropriate or necessary (i.e., if the patient partner does not wish to receive compensation) also help to address this barrier [13].

#### Compensation not prioritized in research budgets

This barrier is related to some researchers and others on the research team or within institutions feeling that providing patient partners with compensation diverts limited budget resources from the items they feel are needed to accomplish the research project (e.g. research personnel, equipment, consumables, etc.). Despite some change, in many instances there is still a deeply engrained belief of volunteerism within the research and healthcare communities when it comes to patients ‘giving back,’ which is one of the reasons patient partners are often not offered payment for their roles on research teams. Sometimes the budget for patient partner compensation is so low that it may appear more tokenistic to patient partners than not being offered compensation at all [28]. These approaches do not recognize the fact that patient partners are crucial, especially in patient-oriented research where patient partners are engaged throughout the research process to reach desired end goals and for their involvement and contributions to integrated knowledge translation [29]. Expecting volunteerism further supports the power imbalances at play in researcher–patient relationships and does not create the optimal working conditions for engagement. It also makes it more difficult to work with marginalized patient partners from diverse backgrounds who may view payment as critical to being engaged.

Examples that illustrate this barrier are:

- Patient partners not being offered compensation based on the assumption that they are or should be ‘satisfied’ with being asked to be a member of the team. This is supported, in our own experiences, by patient partners often being middle class individuals who have the time and therefore having the means to afford doing the work on a voluntary basis.
- Patient partners not being offered compensation due to an assumption that patient partners are simply ‘giving back.’ For example, some patient partners have had positive experiences in the hospital or in a health care system, and wish to give back to the institution and/or research team. While this is rightly their choice, it is not suitable to assume this is the case for all patient partners, nor should we assume ‘giving back’ should not be compensated.

Patient partners participating on funding review panels may help ensure appropriate budgets are allocated to support patient engagement opportunities, including compensation. We note that not all patient partners will want compensation, however it should be best practice to have the conversation about compensation and actively offer it to patient partners from the outset. If the research and healthcare systems truly cannot afford to engage patient partners with all appropriate supports (which may include compensation), should the ‘systems’ undertake to encourage a patient engagement approach?

#### Leadership hesitancy to create a new system

This barrier refers to a potential lack of courage or willingness to be an innovator and early adopter in this space. This potentially means a willingness to make mistakes and invest the time and energy to move the whole community forward, rather than waiting for someone else to take this on. The research community as a whole sometimes has issues with challenging the status quo when it may require more time, resources, effort, or when not explicitly rewarded for it or mandated to do so.

An example that illustrates this barrier is:

- An institution holding off on creating a policy related to patient partner compensation because they know few policies exist within their broader institution community. The institution not wanting to push the envelope on what is required from their researchers (e.g. paperwork, resources) for fear that this might create a competitive disadvantage, or take more financial resources, than other institutions.

A solution to this may be to convene patient partners and others from different research institutions to innovate collectively. These organizations could develop agreed-upon principles to which their own institutions could then adapt internal processes. We have seen consortia being successful because they created and funded substantial patient and public engagement [30].

#### Culture of research teams

This barrier refers to the research culture shift that is slowly occurring to include patient partners on research teams and bring perspectives not traditionally part of research teams. Apart from specific funding calls where patient partners are required to be part of research teams, within academia it may not be clear what the ‘reward’ is to implement patient partnership. For example, metrics related to academic reward often include quantitative measures such as number of publications, conference presentations, and trainees; and amount of grant funding acquired, etc. Despite calls to consider aspects of research quality, including patient engagement, the reality is that these quality measures are not yet norms in research assessment [31, 32]. Given this context, patient partnership may even negatively impact researchers given that the metrics they are typically evaluated on do not acknowledge the value, expertise, and time to do this type of research well.

Examples that illustrate this barrier are:

- Patient partners are only included in studies where patient involvement in research is explicitly promoted in the funding call. Some researchers do not think of patient involvement as a default approach that adds value and incorporate this approach in a tokenistic way to meet basic grant requirements (i.e., to ‘tick a box’).
- Patient engagement specialists, consultants, or other patient engagement roles are only sometimes filled by those who identify as patient partners. It appears there may be a hesitancy to hire individuals who identify as patient partners in to these roles at times.

A solution to this barrier includes incorporating patient partnership into organizations’ strategic goals, hiring patient partners in health care leadership roles, as well as including patient partnership and engagement as part of training and continuing education for researchers and as a recognized and rewarded professional activity [33–35]. Efforts can also be made to involve patient partnership in graduate schools where it is a mandatory component of the curriculum or part of a learning opportunity where patients partners are matched with trainees. Research funding and support for early and meaningful partnership development and engagement is valuable and currently not widespread [36].

#### Preconceived beliefs about the skills and abilities of patient partners

This barrier relates to how much the label of patient in and of itself can change beliefs about their abilities, skills, knowledge, life circumstances, etc. [37, 38]. Patients are often viewed only as voluntary participants in research or health care as opposed to active partners or contributors to it. These beliefs can be held from a variety of view points in the research community, including researchers, administrative and finance staff, research ethics boards members, and others. Patient partners are often viewed as providing only the ‘slice’ of their life that they bring that relates to their patient experiences, when in fact many patient partners have a variety of skills, backgrounds and experiences that they contribute to projects as team members. These all contribute to their lived expertise and experiences, which are frankly not able to be parsed out from their ‘patient only’ experiences.

Some examples that illustrate this barrier include:

- Specific comments from peer reviewers on grant applications about patient partners who have post-secondary education or who have a budget allocated for their compensation as not being ‘real patient partners.’ These comments suggest that somehow these patient partners’ experience in research and healthcare or their education seems to somewhat negate their lived experiences that they would bring to the research team.
- A lack of understanding of the various ways and extents patient research partners may be engaged on a research team. For example, some patient partners play different roles in research, such as knowledge broker, patient engagement facilitator, and even leadership roles. Many contribute actively to research activities, like creating recruitment materials, videos, plain language summaries, linking with other patient partners or patient organizations and more.
- Researchers who also live with chronic diseases and who bring up their experiences in a research setting being critiqued for doing so or choosing not to do so because of the criticism it brings about their potential ‘dual role.’

While it may not be a simple solution, part of a solution is education to support the recognition that patients are people who lead full lives, and whose experience in the healthcare system or disease is simply one part of who they are and how they identify. We need to normalize that patient partners bring their whole selves to research teams, and this may include their skills, professional qualifications, and other aspects of their lives. Supporting patient partners in bringing their entire skill set creates a safe space and environment for collaboration. When the narrow lens on who or what constitutes a patient is brought into a discussion, those who are part of the discussion need to recognize that this is not an appropriate way to talk about or view patients and their experiences.

#### Expectations placed on patient partners

This barrier relates to the expectations that are placed on patient partners without often considering their individual circumstances or what is being asked of them. Patient partners often share very vulnerable experiences and situations or make themselves vulnerable or subject to repeat trauma when they share their experiences. They often agree to requests and deliver under conditions that would not be expected of other members of the research team or that other members of the research team would feel comfortable declining.

Some examples that illustrate this barrier include:

- Attending conferences or meetings for which their expenses are paid, but not their time. In fact, patient partners who work often take time off work or take vacation time to participate in conferences, workshops and meetings.
- Sharing intimate personal and emotional details about their health and healthcare experiences without access to potential support for the related consequences of doing so.
- Contributing to research grant applications, generally without any compensation, under tight timelines while navigating difficult software submission systems.

People who are part of the research and healthcare enterprises need to be more accommodating and thoughtful about how patient partners are engaged. The inherent vulnerability of patients needs to be recognized, as individuals who often bear additional financial and social strains, and experience difficult and often traumatic health crises. These conversations should occur with patient partners at the beginning of the collaboration. Developing institutional policies and support resources that are part of a short onboarding for researchers and patient partners would provide all parties with basic knowledge on the rights of patient partners and responsibilities of researchers to support these.

### Reflection

**Fig. 2:**
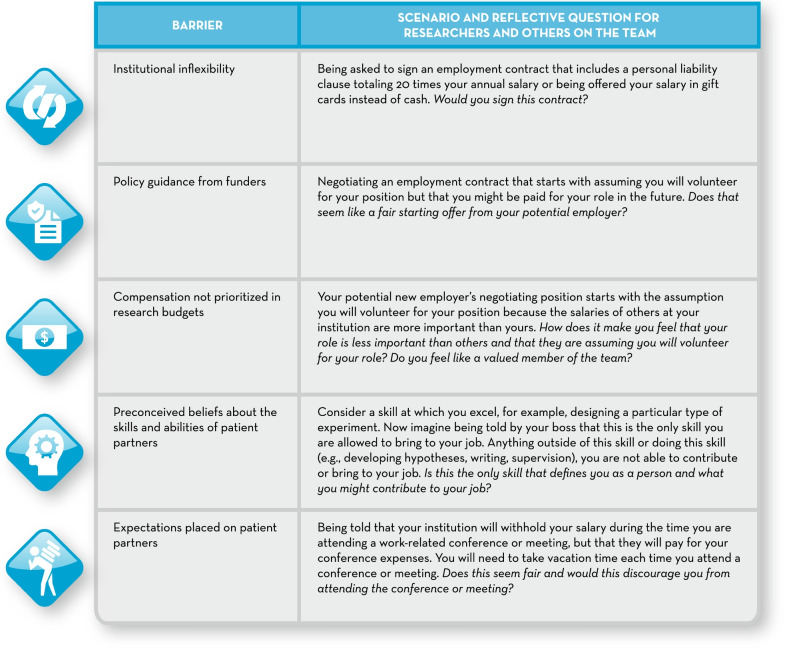
Real-life parallel scenarios. How would you respond? We have created some real-life parallel scenarios to illustrate some of these barriers and the ‘ask’ of patient partners. We ask that you consider how you might respond to these situations

It may be difficult to know exactly how a patient partner feels about a situation involving these barriers to their compensation if you are not a patient partner yourself. We share some real-life scenarios that patient partners have faced in Fig. 2. We ask the reader to reflect on these scenarios and the questions posed. Would you acquiesce to these requests? Why are patient partners asked to do so? What changes are needed? The intention is to illustrate, through empathy or putting yourself in someone else’s shoes, how we might all do better with respect to institutional barriers related to patient partner compensation.

### A call to action

For those working in the area of patient partnership, it is likely that these barriers resonate with their experiences in some way. These barriers impede projects and progress to moving research and healthcare agendas forward with patient partners—diverting individuals and their attention and energy to ‘paperwork.’ There are a number of ways we envision the patient partnership community addressing these barriers. This might be at an individual level or small level changes in the local environment such as sharing this publication and highlighting some of the resources that are included in this publication to colleagues and collaborators. These changes might be accomplished at a higher level such as working to develop and share institutional policies that overcome these barriers.

Our intent in writing this paper is to shine a bright line on these eight institutional barriers to patient partner compensation, provide some solutions and resources to address them, and prompt creation of new ideas so the patient partnership global community can act. In addition to encouraging the community to publish and share good practice examples (on social media, please use #PatientCompensation and/or #PaymentForPPI), we ask you to contact us with how you have acted so that we can share these actions on a patient engagement resource page on the Canadian Institutes of Health Research’s Institute of Musculoskeletal Health and Arthritis’s website [39].

## Conclusions

We presented eight institutional barriers to patient partner compensation based on real-life examples. There are feasible solutions. These may not be the only barriers and potential solutions, but our intention is to start a dialogue on this topic. We encourage the global patient engagement community to consider the importance of overcoming these barriers for both patient partners and researchers so that all members of this community can focus their energies on what they are really passionate about—working together to improve health outcomes.

## Data Availability

Not applicable.
